# Ensemble learning based on efficient features combination can predict the outcome of recurrence-free survival in patients with hepatocellular carcinoma within three years after surgery

**DOI:** 10.3389/fonc.2022.1019009

**Published:** 2022-11-10

**Authors:** Liyang Wang, Meilong Wu, Chengzhan Zhu, Rui Li, Shiyun Bao, Shizhong Yang, Jiahong Dong

**Affiliations:** ^1^ School of Clinical Medicine, Tsinghua University, Beijing, China; ^2^ Division of Hepatobiliary and Pancreas Surgery, Department of General Surgery, Shenzhen People’s Hospital (The Second Clinical Medical College, Jinan University; The First Affiliated Hospital, Southern University of Science and Technology), Shenzhen, Guangdong, China; ^3^ Department of Pediatric Surgery, The Affiliated Hospital of Qingdao University, Qingdao, China; ^4^ Hepato-pancreato-biliary Center, Beijing Tsinghua Changgung Hospital, School of Clinical Medicine, Tsing-hua University, Beijing, China

**Keywords:** recurrence prediction, efficient features, ensemble learning, hepatocellular carcinoma, surgery

## Abstract

Preoperative prediction of recurrence outcome in hepatocellular carcinoma (HCC) facilitates physicians’ clinical decision-making. Preoperative imaging and related clinical baseline data of patients are valuable for evaluating prognosis. With the widespread application of machine learning techniques, the present study proposed the ensemble learning method based on efficient feature representations to predict recurrence outcomes within three years after surgery. Radiomics features during arterial phase (AP) and clinical data were selected for training the ensemble models. In order to improve the efficiency of the process, the lesion area was automatically segmented by 3D U-Net. It was found that the mIoU of the segmentation model was 0.8874, and the Light Gradient Boosting Machine (LightGBM) was the most superior, with an average accuracy of 0.7600, a recall of 0.7673, a F_1_ score of 0.7553, and an AUC of 0.8338 when inputting radiomics features during AP and clinical baseline indicators. Studies have shown that the proposed strategy can relatively accurately predict the recurrence outcome within three years, which is helpful for physicians to evaluate individual patients before surgery.

## 1 Introduction

Hepatocellular carcinoma (HCC) accounts for 85%-90% of the main pathological types of primary liver cancer (1–3). It is easy to spread in the liver through the portal vein system to form intrahepatic metastasis, and it is also easy to form tumor thrombus in the portal vein and cause portal hypertension. HCC is mostly found in the middle and late stages, which leads to its generally poor prognosis (4–8). According to statistics, the recurrence rate of HCC after surgery is as high as about 70% (9), and the survival rate is only 15%-40% (10). Fortunately, treatment modalities represented by precision surgery have greatly improved patient prognosis. Liver resection with early diagnosis can improve the survival rate of patients within one year to 91%-98% (11, 12). Therefore, rational clinical decision-making is essential to reduce recurrence and improve survival.

Accurate preoperative prediction of recurrence can help doctors assess the necessity and risk of surgery, so that they can design rational clinical decisions. Early (1-2 years after surgery) (13) and long-term (5 years and beyond) (14) recurrence predictions have been performed in a small number of studies, with encouraging results. It is worth noting that the recurrence rate of HCC within 3 years after surgery is 50-55%, which accounts for about 71%-78% of the total recurrence (15). Three years after surgery is a critical period, and the absence of recurrence within 3 years indicates a relatively good prognosis. There is no doubt that preoperative prediction of the recurrence outcome in patients within 3 years after surgery is also of great significance for evaluating the illness and selecting treatment options.

The rise of artificial intelligence (AI) technology has brought new strategies for the prediction of HCC recurrence, especially novel data processing methods represented by machine learning and radiomics. Studies have shown that patients’ preoperative imaging, personal information and clinical manifestations are closely related to prognosis (16, 17). Because of this, some researchers have employed the preoperative performance of patients to predict postoperative recurrence through AI algorithms. Ji et al. (18) collected data on 480 patients undergoing HCC resection from 3 centers. Combined with radiomics characteristics and some biochemical indicators, a Cox-based recurrence risk prediction model was constructed, and the final C-index reached 0.633-0.699. Zeng et al. developed a random survival forest (RSF) model using the 15 characteristics of HCC patients. The model obtained a C-index of 0.725 on the validation set, which was encouraging. Huang et al. (19) developed a machine learning prognostic model to identify high-risk patients after surgical resection. The results show that the eXtreme Gradient Boosting tree (XGBoost) achieved the best discrimination in the internal validation queue. In reference (20), 143 features were extracted, including 26 preoperative clinical features, 5 postoperative pathological features, and 112 imaging features, for predicting early recurrence of HCC. As a result, the area under the receiver operating characteristic curve (AUC) of the preoperative model was 0.739, with relatively strong generalization ability.

Nevertheless, there is still room for improvement in the current related work. For example, the lesion area adopted to extract features in most studies needs to be manually segmented from the original image, which brings great challenges to improving work efficiency and reducing costs. In addition, the features of the input model are often not concise and efficient, which will lead to a decrease in accuracy. It is necessary to explore efficient feature representations and achieve automatic and accurate predictions.

This study aimed to develop an excellent predictive strategy for recurrence-free survival (RFS) outcomes in patients with HCC within 3 years after surgery. A 3-dimension deep learning framework was applied to automate lesion segmentation. Seven feature representation methods were compared to explore the most superior feature combinations, including clinical baseline indicators, radiomics features during arterial phase (AP), portal venous phase (PVP), and delayed phase (DP), and combination of clinical data with radiomics features during each phase. Four novel Boosting ensemble learning models were selected for prediction of recurrence outcome. This work has the following highlights:

● Deep learning was employed for automatic segmentation of regions of interest (ROI), which avoided the drawbacks of manual delineation.
● Seven feature representations were explored to find the best model input.
● The study compared novel Boosting ensemble learning methods to select the model with best performance, which may be applicable in the future.

## 2 Materials and methods

**Figure 1:**
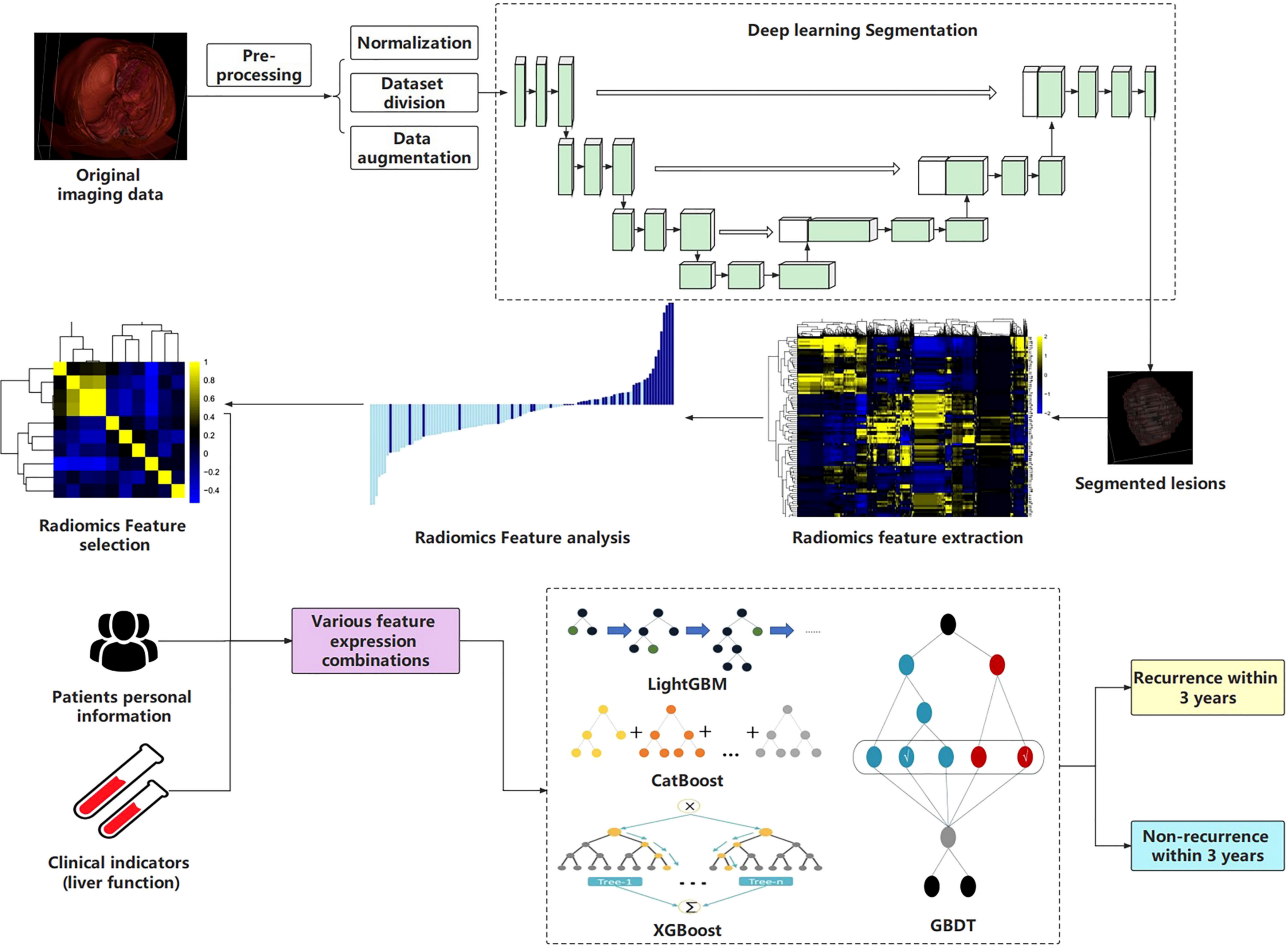
The workflow of this study.

The workflow of this study is shown in **Figure 1**.

### 2.1 Patients

HCC patients who underwent partial hepatectomy in Qingdao University Affiliated Hospital from January 2014 to December 2018 were followed after surgery regularly. The inclusion criteria were as follows: 1. The pathological diagnosis was HCC; 2. The first treatment was partial hepatectomy; 3. Enhanced CT examination was performed within 1 month before surgery, and all periods were completed; 4. The patient’s personal information and relevant clinical data were complete; 5. It has been confirmed that whether the recurrence occurred within 36 months after surgery. The following were the exclusion criteria: 1. Patients who have received chemotherapy, interventional therapy, targeted therapy, etc. before partial hepatectomy; 2. Patients with a history of other tumors; 3. Patients whose tumors have metastasized; 4. Imaging and clinical data were incomplete; 5. The follow-up data were incomplete or the recurrence outcome within 3 years couldn’t be judged. Additionally, all patients included in the study underwent radical hepatectomy. The criteria for radical hepatectomy were: (1) no residual tumor was found at the margin of resection, which was negative; (2) no tumor was found in the remaining liver; (3) tumor markers returned to normal within two months after surgery. Ultimately, 105 patients were selected for the study. RFS period is defined as the time from the date of liver resection to the date of recurrence and within 3 years after surgery is within 36 months from the date of liver resection.

It must be emphasized that the principles of the Declaration of Helsinki were followed and the study was approved by the hospital ethics committee (ethics number: 20001-01). All patients signed an informed consent certificate before surgery.

### 2.2 Imaging acquisition

The scanning equipment for the detection was the German CT (SOMATOM Definition Flash, Siemens) and the American Discovery CT (GE Healthcare). The scanning method was a three-level contrast-enhanced scan of the upper abdomen, and the scanning range was from the top of the liver to the lower edge of the two kidneys. During the scanning process, the voltage, current, scanning layer thickness, layer spacing, and pixel matrix size were set to 120 kV, 200-350 mA, 5 mm, 5 mm, and 512 × 512, respectively. Workers administered iohexol and 350 mg/m1 of iodine through a peripheral vein at a flow rate of 3.0 ml/s and a dose of 1.5 ml/kg under the action of a pressure syringe. Finally, AP, PVP, and DP images were obtained for the study.

### 2.3 Lesion segmentation

Generally, studies mostly segment lesions manually, which reduces work efficiency. Based on the previous manual annotation, we built a 3D U-Net deep learning model for automatic and accurate segmentation of lesions.

#### 2.3.1 Manual annotation

This work adopted the supervised learning to automatically segment the ROIs, so manual annotation was required before model training. Two physicians with extensive experience in radiology were selected for this task, one of whom delineated the tumor area of each slice with the help of 3D Slicer (Boston, MA, USA) software without knowing any patient’s baseline data, and the other one was responsible for checking the annotation results. Once there was a dispute, return to discuss and re-mark if necessary. All CT images for the three periods were delineated and formed into volumes of interest (VOIs).

#### 2.3.2 Data pre-processing

Considering that some slices in CT images do not contain ROIs, this will increase the computational complexity. Slices without lesions were cropped according to the annotated images and the remainders were studied. Moreover, we normalized the image format to 256×256×48 for better input to the model. In order to expand the amount of data, data augmentation operations were performed on the divided training set, including but not limited to image flipping, rotation, cropping, scaling, and blurring (21, 22).

#### 2.3.3 Construction of segmentation model

CT images have 3D structures, and the traditional method convert them into 2D slices and then send into the 2D segmentation model, which results in the loss of spatial information. In this study, a 3D convolutional neural network (3D U-Net) was constructed to segment lesions directly, which comprehensively preserved the spatial information between slices (23, 24).

Similar to the classic U-Net, the 3D U-Net also consists of Encoder and Decoder, each of which contains four sub-modules. In the Encoder, each sub-module contains two 3 × 3 × 3 convolutional layers, and each convolutional layer is connected to an activation function. After completing the convolution operation, max-pooling with a stride of 2 is performed on each dimension. In Decoder, each sub-module contains an upsampling process (deconvolution operation) with a stride of 2, and then two 3 × 3 × 3 convolutional layers and activation functions are added in turn. It must be emphasized that the padding in the convolutional layer of this module is set to 1, which makes the convolution operation not change the size of the image. Changes in image size are completely controlled by pooling and upsampling. Additionally, the last sub-module of the Decoder consists of a 1 × 1 × 1 convolutional layer, which reduces the number of output feature maps. Batch normalization (BN) was introduced before each activation function.

This work aims to segment liver tumors from other tissues, where the input channel of the model was set to 256 × 256 × 48, and the activation function adopted ReLU. After the construction was completed, the total parameters and the trainable parameters of the neural network reached 4,122,466 and 4,117,570, respectively.

### 2.4 Radiomics feature extraction and selection

Feature extraction is an essential part of radiomics analysis. In this study, we performed radiomic feature extraction for segmented liver tumors. Using the Pyradiomics 3.0.1 library in Python, a total of 788 dimensional features including Shape, Firstorder, GLCM, GLRLM, GLSZM, and GLDM were extracted. Each type of features was performed 9 transformations including Original, Wavelet-LLH, Wavelet-LHL, Wavelet-LHH, Wavelet-HLL, Wavelet-HLH, Wavelet-HHL, Wavelet-HHH, and Wavelet-LLL. Among them, “Wavelet-XXX” represents the wavelet transform, followed by the corresponding basis function type.

Due to the high dimension of the extracted features, it is easy to cause “dimensionality disaster” and affect the model performance. Therefore, selecting features with large contributions can reduce the dimension as much as possible without affecting the comprehensiveness of the features. This work employed the Least Absolute Shrinkage Selector Operator (Lasso) algorithm to select the extracted features and ranked the contribution of each feature. By constructing a penalty function, Lasso can compress the coefficients of variables and make some regression coefficients 0, so as to achieve the purpose of variable selection. In addition, Lasso can also filter variables and reduce the complexity of the model. The variable screening here refers to not putting all the variables into the model for fitting, but selectively putting the variables into the model to get better performance parameters. Complexity adjustment refers to controlling the complexity of the model through a series of parameters to avoid overfitting. The optimal model was fit and the value of the penalty parameter α was determined based on the sklearn library in Python. For the dimensionality-reduced features, correlation coefficients and cluster heatmaps, as well as the coefficient distribution of each feature are visualized to better interpret the radiomics features.

### 2.5 Selection of clinical baseline features

This study collected clinical baseline data of HCC patients in addition to CT images, such as personal information and clinical indicators. The gender and age of patients were collected as personal information data. Clinical indicators here were mainly tumor markers and liver function indicators, including alpha-fetoprotein (AFP), hepatitis B surface antigen (HBsAg), albumin (ALB), the total bilirubin (T- BIL), alanine aminotransferase (ALT) and aspartate aminotransferase (AST), etc. It should be noted that positive and negative results were obtained for AFP and HBsAg, while other liver function indicators were represented as specific test results.

### 2.6 Construction of recurrence prediction models

A total of seven feature representations, including selected radiomics features during AP, PVP, and DP, clinical baseline features, and their combined features, were input into the recurrence prediction models. The Boosting ensemble learning algorithms were adopted to predict the RFS outcome within 3 years.

#### 2.6.1 Light gradient boosting machine

Gradient Boosting Decision Tree (GBDT) is a classic ensemble algorithm in machine learning. Its main idea is to employ weak classifiers (decision trees) to iteratively train to obtain the optimal model, which has the advantages of good training effect and not easy overfitting. LightGBM (Light Gradient Boosting Machine) is a framework for implementing the GBDT algorithm. It supports efficient parallel training and has faster training speed, lower memory consumption, better accuracy, support for distributed and fast processing of massive data, etc. (25). Currently, this framework has been relatively widely used in the field of medical data processing (26–28), but it has not been attempted in the HCC recurrence prediction task.

A leaf-wise algorithm with a depth limit is adopted in LightGBM. This strategy finds the leaf with the largest split gain from all the current leaves each time, and then splits and loops, which reduce more errors and get better accuracy under the same number of splits. Moreover, the Gradient-based One-Side Sampling (GOSS) operation is proposed to reduce computation and improve accuracy. This method does not calculate the gradient through the sample points used, but calculates the gradient by partial sampling. The Exclusive Feature Bundling (EFB) is also proposed to bundle some features together to reduce the feature dimension, thereby reducing the time-consuming to find the best fork. This study implemented the LightGBM algorithm based on the sklearn library in Python to perform the binary classification task, that is, recurrence or not within 3 years.

#### 2.6.2 Categorical boosting

Categorical Boosting (CatBoost), as a novel ensemble learning algorithm, has been applied to some medical data processing tasks, but has not been used to predict HCC recurrence (29, 30). Catboost adopts the oblivious tree as the base tree model, which is characterized by the same segmentation features in each layer. Leaf nodes can be converted to binary codes, and the value of the node is stored in a floating-point vector of length 2 to the power of *d* (*d* is the depth of the tree). One of the advantages of this tree is that the prediction performance is better, and this structure can also weaken the shortcomings of easy fitting in decision trees to a certain extent. When Catboost completes training, it stores the leaf node value of each tree into a vector. When predicting, it can quickly retrieve the corresponding leaf node value by judging which leaf node it is in, so it can improve the prediction efficiency and enhance the model performance. This work selected it for predicting HCC recurrence.

#### 2.6.3 eXtreme gradient boosting

XGBoost has been widely used in the field of medical data analysis since it was proposed in 2014 (31, 32). In the HCC recurrence prediction task, this algorithm was also tried and achieved significant results (19). Its greedy algorithm-based split node calculation and missing value handling techniques are very suitable for data mining. The algorithm was trained to predict RFS outcomes and compared with other models such as LightGBM and CatBoost.

#### 2.6.4 Gradient boosting decision tree

We also employed GBDT as the baseline model for comparison. It is an ensemble learning algorithm based on decision trees that iterates over new learners through gradient descent. In this paper, the classification task was performed, and the Classification And Regression Tree (CART) was selected.

### 2.7 Statistical analysis

For the analysis of clinical baseline data, the differences involved in this study were compared using student t-test or Mann-Whitney U-test, where the criterion of significant difference was set at P<0.05. Mean ± 95% confidence interval (CI) was calculated as results for continuous variables. To reflect the criticality of certain variables, the univariate Kaplan-Meier curve was introduced for survival analysis.

We calculated the mean Intersection overUnion (mIoU), accuracy (Acc), Kappa and Dice coefficients of 3D U-Net to reflect the segmentation effect. Additionally, Acc, recall, precision (Prec), F_1_ score, receiver operating characteristic curve (ROC) and corresponding AUC were introduced as performance evaluation criteria for the ensemble learning models. It should be emphasized that the classification threshold was set to 0.5.

### 2.8 Experimental setup

The image data during the three scanning periods were randomly divided into training set, validation set and test set according to the ratio of 8:1:1. The segmentation model was trained on the training set and validation set, and the test set was employed to demonstrate the performance. All lesions segmented by the model during three periods were acquired and their radiomic features were extracted. For the Lasso regression algorithm, the study obtained the best α value through 10-fold cross-validation to select key features. Considering the small sample size, this study selected the 5-fold cross-validation method to determine the features representation and predict the recurrence outcome, and calculated the mean value of five experiments and the corresponding 95% CI as the results. The relevant computing equipment for this experiment was configured with a CPU AMD Ryzen 7 5800H (16 GB memory) and a GPU NVIDIA^®^ Tesla V100 (32 GB memory) with acceleration support of the compute unified device architecture (CUDA). All work was carried out in the Windows 10 operating system, and the programming language, deep learning framework and key libraries included Python 3.7, Pytorch, Pyradiomics, sklearn, VTK, etc.

## 3 Results

### 3.1 Analysis of patients’ basic data

**Figure 2:**
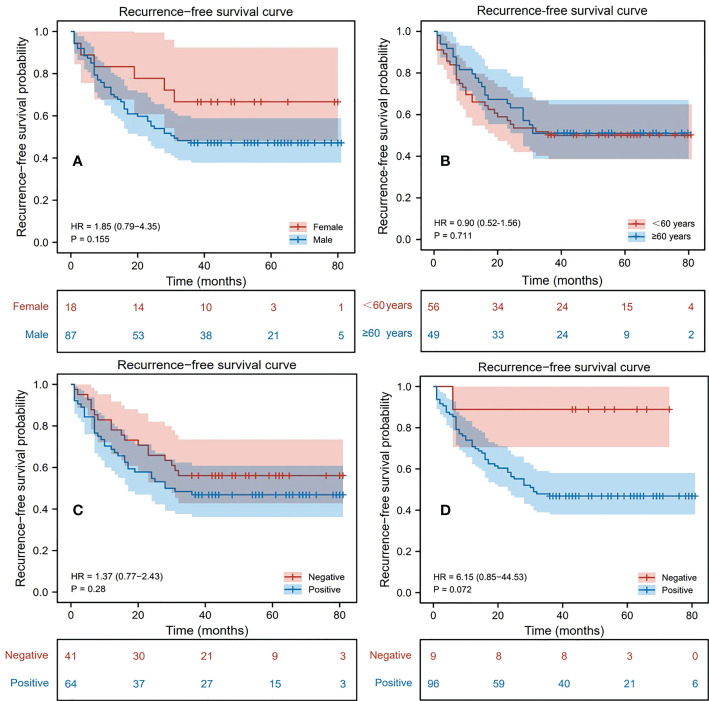
Kaplan-Meier survival analysis curve of patients, where the variables in **(A–D)** are gender, age, alpha-fetoprotein (AFP), hepatitis B surface antigen (HBsAg) respectively.

During follow-up, 52 patients (49.52%) were found to have recurrence within 3 years after surgery, of which 46 (88.46%) were male and 6 (11.54%) were female; 24 (46.15%) were aged 60 years or older and 28 (53.85%) were younger than 60 years old; 34 (65.38%) were AFP positive, and 18 (34.62%) were negative; 51 (98.08%) were HBsAg positive, and 1 (1.92%) were negative. 53 patients (50.48%) were found to have no recurrence within 3 years after surgery, of which 41 (77.36%) were male and 12 (22.64%) were female; 25 (47.17%) were aged 60 years or order and 28 (52.83%) were younger than 60 years old; 30 (56.60%) were AFP positive, and 23 (43.40%) were negative; 45 (84.91%) were HBsAg positive, and 8 (15.09%) were negative. Based on this, a univariate Cox proportional hazards model was established to judge the influence of different factors on RFS, and the related results were represented by the Kaplan-Meier curves (**Figure 2**). Through the statistics of gender classification group (HR=1.85, P=0.155) and HBsAg result classification group (HR=6.15, P=0.072), it was found that gender and HBsAg affect RFS to some extent although the differences were not significant, followed by AFP (HR=1.37, P=0.280). Notably, age was not significantly associated with recurrence outcome from the age-categorized group in this study (HR=0.90, P=0.711). However, patient’s age is a key factor affecting prognosis from previous studies (20, 33), so we still regarded it as one of the features. **Table 1** shows the statistical results of some continuous clinical indicators. It can be found that ALB, T-BIL, ALT, and AST (P=0.149, 0.377, 0.128, and 0.223, respectively) were relatively significantly different or not significantly different between the recurrence and non-recurrence groups.

**Table 1:** Statistical results of 4 clinical indicators.

Clinicalindicator	Total dataset (*N* = 105)		P-value
	Recurrence (*N* =52)	Non-recurrence (*N* = 53)	
ALB(g/L)	40.36 ± 1.28	40.42 ± 1.26	0.1493
T-BIL(mmol/L)	20.32 ± 4.81	20.34 ± 4.69	0.3765
ALT(u/L)	51.12 ± 15.42	50.89 ± 15.02	0.1282
AST(u/L)	42.78 ± 12.23	42.20 ± 11.92	0.2233

all outcomes are based on recurrence within 3 years after surgery. ALB, T-BIL, ALT, and AST represent albumin, the total bilirubin, alanine aminotransferase and aspartate aminotransferase, respectively. Each indicator is represented by the mean of the sample and the corresponding 95% confidence interval (CI).

### 3.2 Results of lesion segmentation

**Figure 3:**
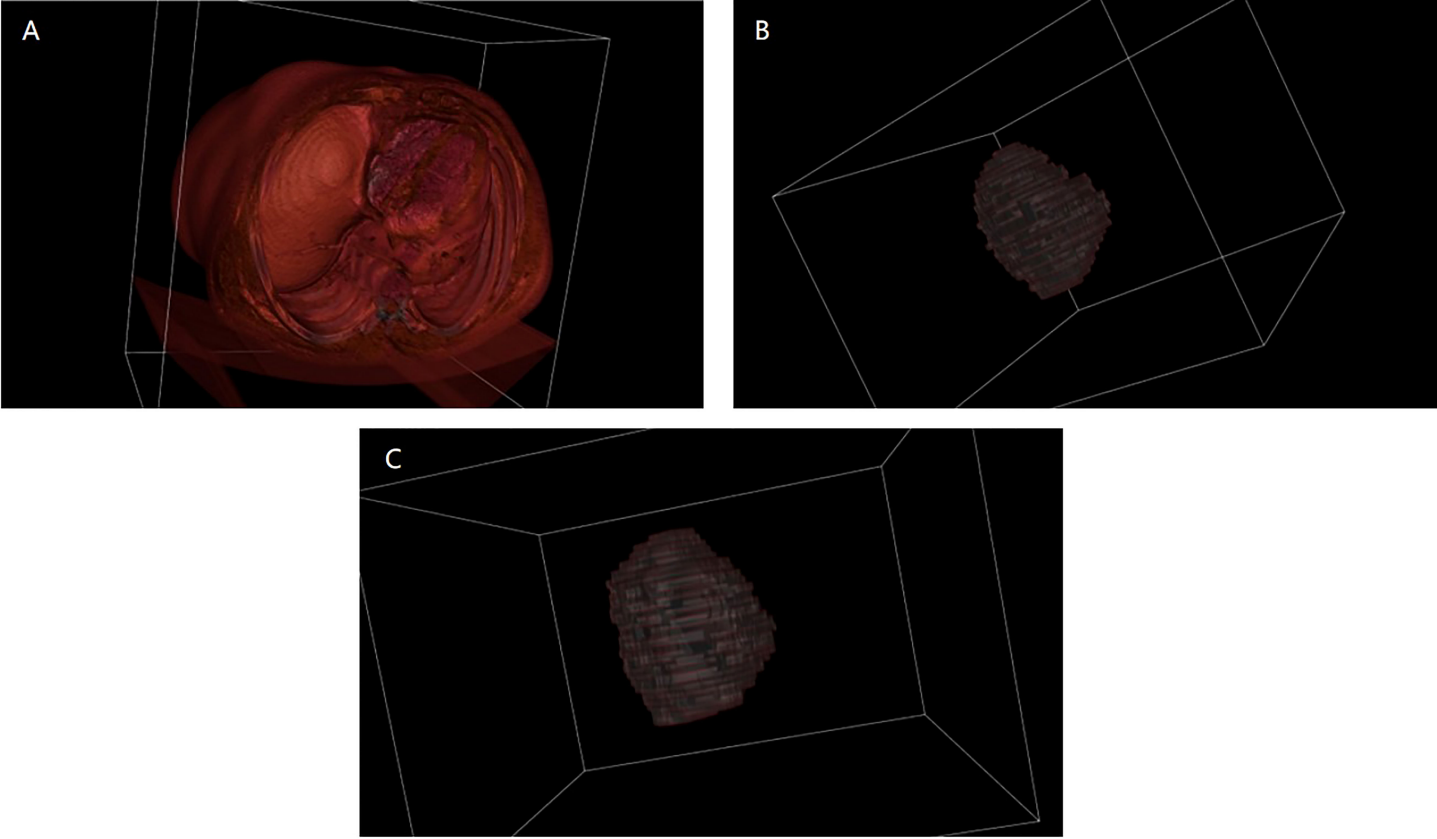
3D reconstruction visualization images before and after segmentation. **(A)** is the 3D visualization of the original CT image before segmentation; **(B)** is the 3D visualization after manually segmenting the tumor; **(C)** is the 3D visualization after segmenting the tumor using deep learning.

The training and validation sets during the three periods were input into 3D U-Net for training, and the model performance was optimized through parameter adjustment and continuous iteration. The key hyperparameters were set as follows: Momentum optimizer was selected and set to 0.9, initial learning rate, weight_decay and batch_size were set to 0.001, 4.0×10^-3^, 2, respectively. After the model iterated for 500 epochs, it fully converged (the loss value of the validation set was lower than 0.001). At this point, we stopped the training and saved the parameters. The performance on the test set was excellent, with mIoU of 0.8874, Acc of 0.9915, Kappa of 0.8738 and Dice coefficient of 0.9360, which indicates that the deep learning model has strong generalization ability for segmenting liver lesions. To visually compare the segmentation effects, this paper presents 3D reconstruction visualization images of the upper abdomen based on CT scans, manually annotated tumors, and deep learning-segmented tumors (**Figure 3**). The VTK library in Python was adopted as the relevant drawing tool. It must be emphasized that the lesion areas involved in subsequent calculations were automatically segmented by the trained model.

### 3.3 Results of radiomics feature extraction and selection

A total of 788 radiomic features were extracted in this study, including 100 features from original transform and 688 features from wavelet transform. In the original transform, the extracted contents were 14 shapes, 18 firstorder, 22 GLCM, 16 GLRLM, 16 GLSZM and 14 GLDM features. In the wavelet transform, the contents extracted by Wavelet-LLH, Wavelet-LHL, Wavelet-LHH, Wavelet-HLL, Wavelet-HLH, Wavelet-HHL, Wavelet-HHH, and Wavelet-LLL included 144 firstorder, 176 GLCM, 128 GLRLM, 128 GLSZM and 112 GLDM features. Since high-dimensional features may affect model performance, dimensionality reduction and selection of contributing features is significant.

**Figure 4:**
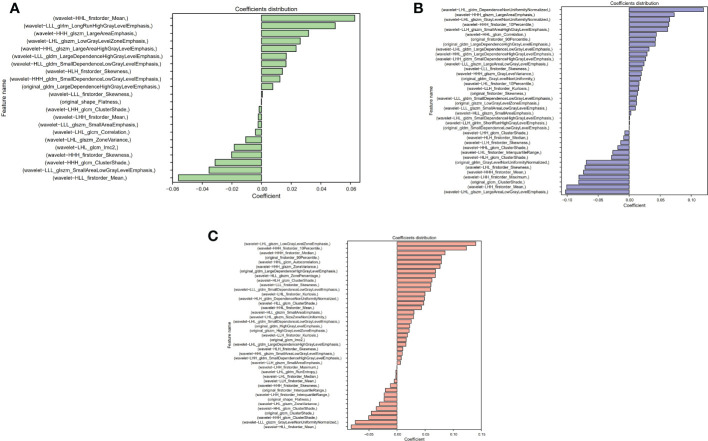
Distribution of selected radiomics feature coefficients. **(A–C)** show the features and their distributions during arterial phase (AP), portal venous phase (PVP) and delay period (DP), respectively.

**Figure 5:**
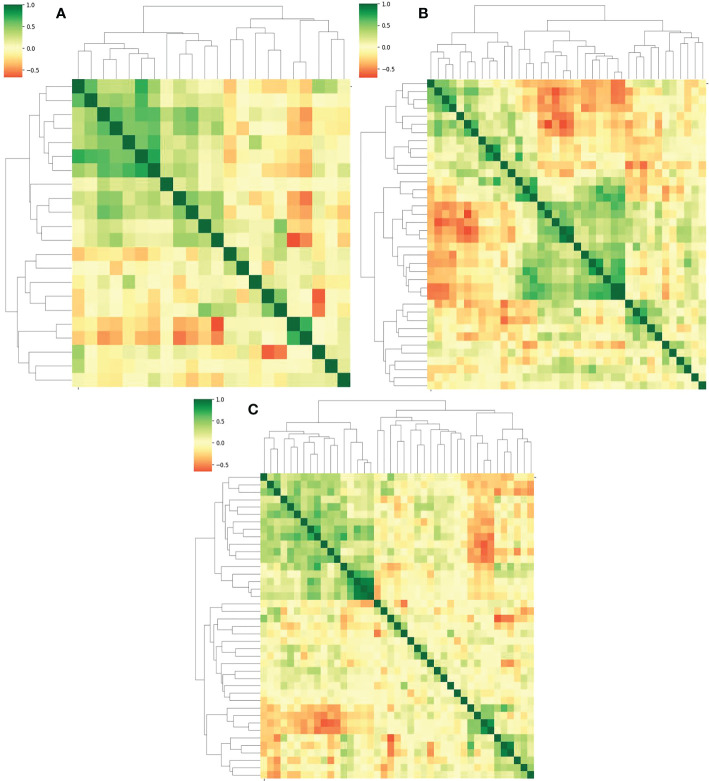
**(A–C)** represent the correlation and the clustering heatmaps between features during the AP, PVP and DP, respectively.

The Lasso algorithm was used for fitting to obtain the best α values during AP, PVP and DP, respectively. The model was fully converged after 10,000 iterations based on the 10-fold cross-validation. The optimized α values for AP, PVP and DP were calculated as 0.0518, 0.0244 and 0.0202, respectively. Meanwhile, 22, 38, and 41 features with contribution degrees were selected during the above three periods respectively. **Figures 4A, B**, and C show the selected feature names and the corresponding coefficients distribution in AP, PVP, and DP, respectively. **Figure 5** shows the correlation coefficient between the features and the clustering results through heatmaps (the color depth represents the correlation strength).

### 3.4 Results of recurrence prediction

#### 3.4.1 Comparison of different feature representations

**Figure 6:**
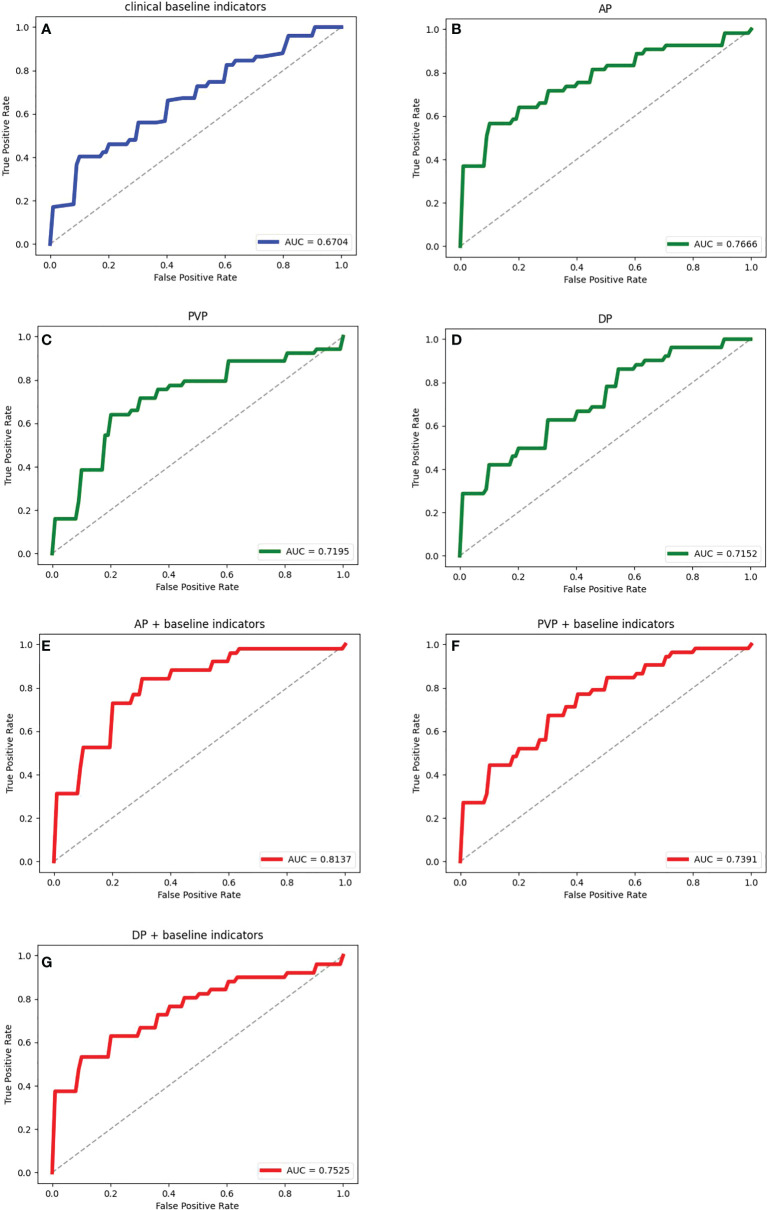
The ROC curves and the corresponding AUCs of the ensemble learning model with different feature representations. **(A)** is the result of inputting personal information and clinical indicators; **(B–D)** are the results of inputting radiomic features during AP, PVP and DP respectively; **(E–G)** are the results of inputting radiomics features during AP, PVP and DP combined with clinical data respectively.

Seven feature representation methods for evaluating the prognosis of HCC were considered, including clinical baseline features, radiomics features of AP, radiomics features of PVP, radiomics features of DP, radiomics features of AP combined with clinical indicators, radiomics features of PVP combined with clinical indicators, and radiomics features of DP combined with clinical indicators. In order to explore the most excellent feature representation, we separately input the above features into the ensemble learning algorithms and optimized the training. Considering the randomness of the results based on the small sample size, the training process adopted 5-fold cross-validation, that is, the dataset was randomly divided into 5 equal parts, 4 of which were used for training and the remaining 1 was used for testing. This step was repeated 5 times. The average value of 5 experiments and the corresponding 95% CI were regarded as the evaluation standard. Meanwhile, the ROC curves and their AUC values reflected the generalization ability of the models. The ROC curves of the models with different features were drawn and their AUC values were calculated. Due to space limitations, we only show the results using the LightGBM algorithm in **Table 2** and **Figure 6**, and the rest of the results are in the Appendix. It can be seen that the effect of combining radiomics features with clinical baseline indicators was better than inputting radiomics features or clinical indicators alone, with AP combining obtaining the best effect, followed by DP combining and PVP combining. The effect of only inputting clinical indicators was the least satisfactory, which might be caused by too little information represented by the features.

**Table 2:** Comparison of recurrence prediction results of ensemble learning models using different feature representations.

Feature representation	Acc	Recall	Prec	F_1_ score
Personal and	0.6062	0.6164	0.6019	0.6039
clinical indicators	± 0.0877	± 0.1429	± 0.0930	± 0.1027
AP	0.7224	0.6946	0.7528	0.7156
	± 0.0834	± 0.0944	± 0.1231	± 0.0804
PVP	0.6438	0.6782	0.6409	0.6570
	± 0.1117	± 0.1122	± 0.1019	± 0.1014
DP	0.6343	0.6909	0.6331	0.6560
	± 0.0690	± 0.0413	± 0.0872	± 0.0520
AP+	0.7495	0.7673	0.7402	0.7502
other indicators	± 0.0629	± 0.1051	± 0.0525	± 0.0710
PVP+	0.6824	0.6927	0.6844	0.6846
other indicators	± 0.0783	± 0.0941	± 0.0780	± 0.0771
DP+	0.6819	0.6309	0.7168	0.6630
other indicators	± 0.0659	± 0.0912	± 0.1273	± 0.0756

Each result is represented by the mean of 5 experiments and 95% CI.

#### 3.4.2 Comparison of prediction models

**Figure 7:**
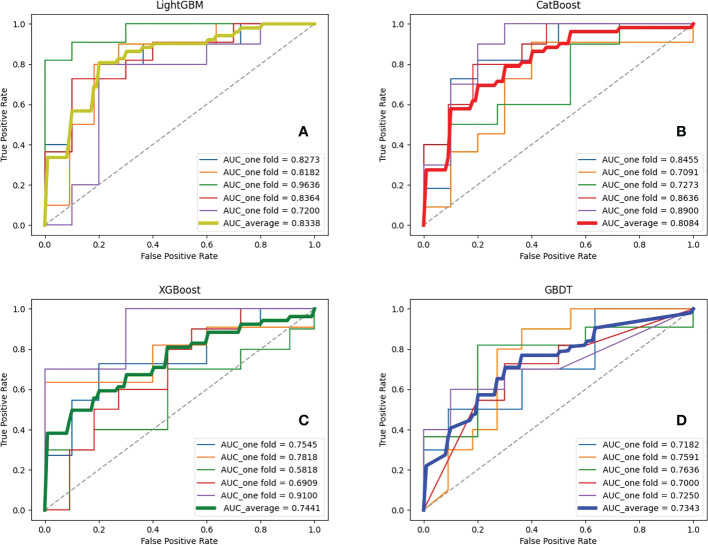
ROC curves and corresponding AUCs of various ensemble learning models. **(A–D)** represent the results of ensemble learning models - Light Gradient Boosting Machine (LightGBM), Categorical Boosting (CatBoost), eXtreme Gradient Boosting (XGBoost) and Gradient Boosting Decision Tree (GBDT), respectively.

Seven feature representations were employed to compare the performance of ensemble learning models. Likewise, the study performed five-fold cross-validation on each model and calculated the associated evaluation metrics. During training, GridSearchCV method was adopted to adjust the model parameters and no overfitting occurred for each model. Due to space limitations, we only show the results inputting the most effective feature representations in this section, and the rest of the results are in the Appendix. It is found that for the four ensemble learning algorithms, different feature expressions input have similar laws, so the following only analyzes the models when radiomics features during AP and clinical indicators are input. **Table 3** shows certain key parameters of each model. The test results of the Boosting ensemble models are shown in **Table 4**. It can be found that the performance of LightGBM was the most excellent, with an average Acc of 0.7600, recall of 0.7673, Prec of 0.7733, and F_1_ score of 0.7553, which indicated that this algorithm can accurately predict recurrence outcome within 3 years after surgery. It is worth noting that XGBoost performed well in previous similar studies, but not as good as the former in this task. It had an Acc of 0.7224 and an F_1_ score of 0.6936, which was not as superior to LightGBM. Additionally, as the baseline model, GBDT only obtained an average Acc of 0.6543, recall of 0.6382, Prec of 0.6600 and F_1_ score of 0.6387. The per-fold and averaged ROC curves and corresponding AUC values are shown in **Figure 7**. LightGBM had the strongest generalization, and its AUC reached 0.8338 (CI: ± 0.0680), followed by CatBoost (0.8084 ± 0.0650), XGBoost (0.7441 ± 0.0946), and GBDT (0.7343 ± 0.0214).

**Table 3:** Key parameter settings for each ensemble learning model.

Model	Parameter name	Parameter settings
LightGBM	n_jobs	-1
	n_estimators	600
	learning_rate	0.01
	max_depth	5
	num_leaves	32
	colsample_bytree	0.51
	subsample	0.6
CatBoost	iterations	5000
	learning_rate	0.01
	l2_leaf_reg	3
	bagging_temperature	1
	subsample	0.6
	random_strength	1
	depth	6
	border_count	128
XGBoost	learning_rate	0.001
	n_estimators	1000
	max_depth	5
	min_child_weight	1
	gamma	0
	subsample	0.6
	colsample_bytree	0.8
	seed	27
GBDT	n_estimators	1000
	learning_rate	0.01
	max_depth	5
	random_state	4

**Table 4:** 5-fold cross-validation results for recurrence prediction using different ensemble learning models.

Model	Acc	Recall	Prec	F_1_ score
LightGBM	0.7600	0.7673	0.7733	0.7553
	± 0.0579	± 0.1311	± 0.0771	± 0.0716
CatBoost	0.6833	0.6164	0.7107	0.6511
	± 0.0543	± 0.1313	± 0.0397	± 0.0879
XGBoost	0.7224	0.6346	0.8032	0.6936
	± 0.0834	± 0.1154	± 0.1521	± 0.0978
GBDT	0.6543	0.6382	0.6600	0.6387
	± 0.0463	± 0.1328	± 0.0286	± 0.0828

Each model was evaluated employing the mean of each fold result and corresponding 95% CI.

## 4 Discussion

In this study, the LightGBM model was constructed for the first time to accurately predict the recurrence outcome of HCC within three years after surgery. An efficient feature representation was explored, that is, the combination of radiomics features of tumor during AP, patient personal information, and clinical indicators. We trained the deep learning automatic segmentation model to make the process efficient. The results show that the proposed method was the most effective, achieving an accuracy of 0.7600 and an AUC of 0.8338.

Compared with manual segmentation, although the effect of deep learning segmentation is not as good as the former, it has higher efficiency and lower labor cost (34). In this paper, the mIoU of 3D U-Net reached 0.8874, which indicated that this algorithm can accurately segment the liver tumor region. It only took 1.22-1.85s to execute each sample on the local device, which was much faster than the manual way. It is undeniable that deep learning with excellent performance is the future trend of lesion segmentation methods (35).

This work selected 22 radiomics features during AP combined with 8 clinical baseline features from the seven feature representations and validated superiority. This feature combination eliminated dimensional redundancy, including tumor features with large contribution coefficients and clinical factors that affect prognosis. Notably, the present study found that the radiomics features during AP were superior to during PVP and DP, suggesting that AP might better capture features affecting recurrence. In addition, combined representations outperformed individual clinical or radiomics feature representations. Possibly the combination increased the amount of available information, making the model more likely to learn complex preoperative-prognostic associations (36).

Four novel Boosting ensemble models were adopted for comparison, among which LightGBM achieves the best performance (AUC=0.8338), outperforming CatBoost (AUC=0.8084), XGBoost (AUC=0.7441) and GBDT (AUC=0.7343) when inputting radiomics features during AP and clinical baseline indicators. Previous studies have confirmed the state-of-the-art of the XGBoost algorithm in the HCC prognosis prediction task (19). XGBoost belongs to the boosting family and is an engineering implementation of the GBDT algorithm. It focuses the residuals during training, uses a second-order Taylor expansion in the objective function and adds regularization. Meanwhile, the exact greedy idea is adopted in the generation process of the decision tree. When looking for the best split point, a pre-sort algorithm is adopted, that is, all features are pre-sorted according to the value of the feature, and then all the split points on all the features are traversed, and the total number of samples split according to these candidate split points is calculated. The objective function gain is to find the feature and candidate splitting point corresponding to the maximum gain, so as to split. XGBoost training is performed by addition, that is, each time a tree is trained by focusing residuals, and the final prediction result is the sum of all trees. However, XGBoost performs pre-sorting in the selection of optimal split points, and then calculates the objective function gain of all samples for all split points of all features. The space and time complexity of this process is very large, and to a certain extent affects the accuracy (31).

To address this issue, we adopted LightGBM for predicting recurrence. Based on XGBoost, LightGBM employs histogram algorithm to solve the problem of excessive number of split points. This method takes up less memory and reduces computation time. Secondly, it introduces the GOSS algorithm, which extracts according to the weight information of the samples to reduce a large number of samples with small gradients, and at the same time does not change the distribution of the dataset too much. Moreover, LightGBM also proposes the EFB mode, which reduces dimensionality by bundling features. Therefore, LightGBM can improve the model accuracy while reducing the computational effort (37), which leads to its better performance in the prognosis prediction task. In the future, it is necessary to further validate the applicability of the proposed method on larger datasets.

It should be emphasized that this study aimed to predict the postoperative recurrence risk of patients only through preoperative factors, including preoperative imaging examination and clinical indicators detection. Because only in this way can it help the doctor’s clinical decision-making. Although postoperative pathological examinations, such as microvascular invasion (MVI) are very meaningful for recurrence prediction (38), they were not considered in this study. The feasibility and effectiveness of this method have been demonstrated in reference (39, 40).

There are some other studies to predict the recurrence of HCC after surgery. Shen et al. (41) used the TCGA database and machine learning method to build a prediction model for recurrence of HCC patients, and optimized the recurrence prediction model. After the model was optimized, the prediction accuracy was 74.19%. Lee et al. (20) employed genetic algorithm to predict early recurrence of HCC, and extracted a total of 143 features, including 26 preoperative clinical features, 5 postoperative pathological features, and 112 imaging features. After training, the AUC of the preoperative and postoperative models were 0.781 and 0.767 on the training set, and 0.739 and 0.741 on the test set, respectively. Saito et al. (42) adopted support vector machine (SVM) to predict the recurrence outcome of HCC patients based on the postoperative pathological results. The patients were grouped according to the criteria of recurrence within 1 year, 1-2 years, and 4 years after resection. The final accuracy of ROI prediction in HCC and non-HCC regions was 80.6% and 68.1%, respectively. It must be emphasized that our work only collected 105 patients, but still obtained relatively remarkable performance, suggesting that the proposed method had more potential for predicting recurrence outcomes.

It is undeniable that the present study still has some shortcomings. For example, the small sample size from a single center challenges the applicability of the models. This work only focuses on the prediction of recurrence outcomes within 3 years, and further follow-up is required to predict at different times in the future. Moreover, the proposed method has not been tested in real clinical practice, which needs to be validated in the future. Zeng et al. (43) developed a machine learning method to predict the early recurrence of radical HCC hepatectomy using the data from two centers, and the effect was relatively significant. While we have mined the key features that influence the model, the interpretability issues of machine learning still need to be addressed.

## 5 Conclusion

This study aims to help physicians to evaluate the effectiveness of surgery and thus facilitate rational clinical decision-making. An ensemble learning strategy based on efficient feature representation was proposed for the recurrence outcome in HCC patients within three years after surgery. The 3D U-Net was used to automatically segment the lesions. Radiomics features during AP and clinical baseline features were selected as input and four ensemble models were trained. The results showed that LighGBM outperformed other ensemble algorithms, suggesting that it may be a novel model for predicting recurrence. In the future, the dataset will be expanded for early and late recurrence prediction and external clinical validation will be performed to validate the applicability of the method. When the generalization ability of the method is successfully verified, the relevant software (or web program) will be designed and applied to clinical practice.

## Data availability statement

The data analyzed in this study is subject to the following licenses/restrictions: Since the dataset involved in this study involves patient privacy and has signed a non-disclosure agreement, it cannot be made public. Requests to access these datasets should be directed to Jiahong Dong, dongjiahong@mail.tsinghua.edu.cn.

## Ethics statement

Written informed consent was obtained from the individual(s) for the publication of any potentially identifiable images or data included in this article.

## Author contributions

LW: Study concept, image preprocessing, experimental design, data analysis, writing of manuscript. MW: Experimental design, editing the manuscript, and data collection. CZ: Data analysis and data collection. RL: Data collection. SB: Experimental design. SY and JD: Study concept and funding. All authors contributed to the article and approved the submitted version.

## Funding

This work was supported by National Natural Science Foundation of China (grant number: 82090052, 82090050, 81930119); CAMS Innovation Fund for Medical Sciences (grant number: 2019-I2M-5-056).

## Conflict of interest

The authors declare that the research was conducted in the absence of any commercial or financial relationships that could be construed as a potential conflict of interest.

## Publisher’s note

All claims expressed in this article are solely those of the authors and do not necessarily represent those of their affiliated organizations, or those of the publisher, the editors and the reviewers. Any product that may be evaluated in this article, or claim that may be made by its manufacturer, is not guaranteed or endorsed by the publisher.
