# Supplementary material for: Ensemble learning based on efficient features combination can predict the outcome of recurrence-free survival in patients with hepatocellular carcinoma within three years after surgery
Source: Front Oncol. 2022 Nov 10;12:1019009. doi: 10.3389/fonc.2022.1019009 (PMC9686395; doi:10.3389/fonc.2022.1019009)
Supplement: Supplementary file 1 [file DataSheet_1.pdf]

Appendix

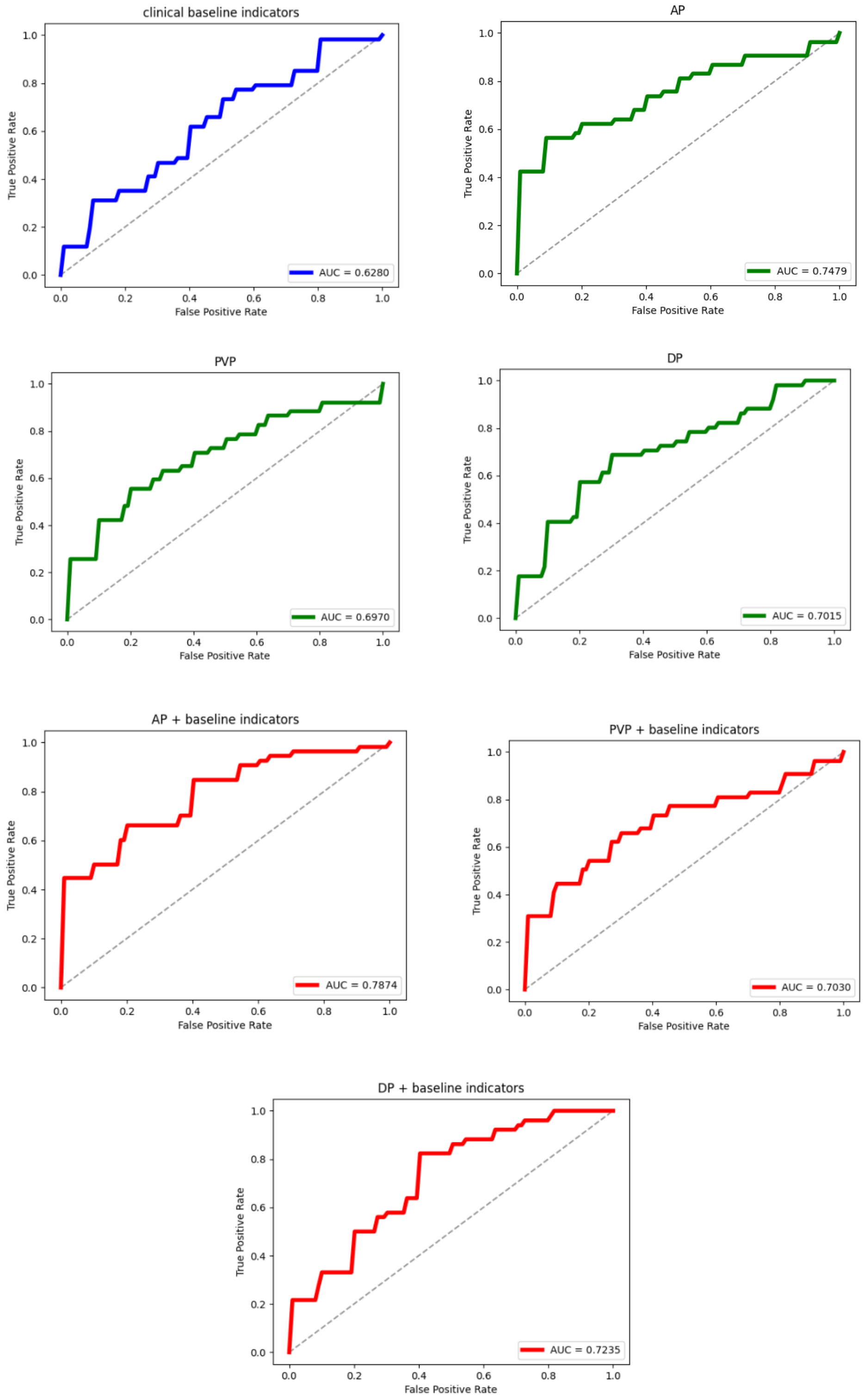

Fig 1: Results of using 7 feature representations in the Catboost model

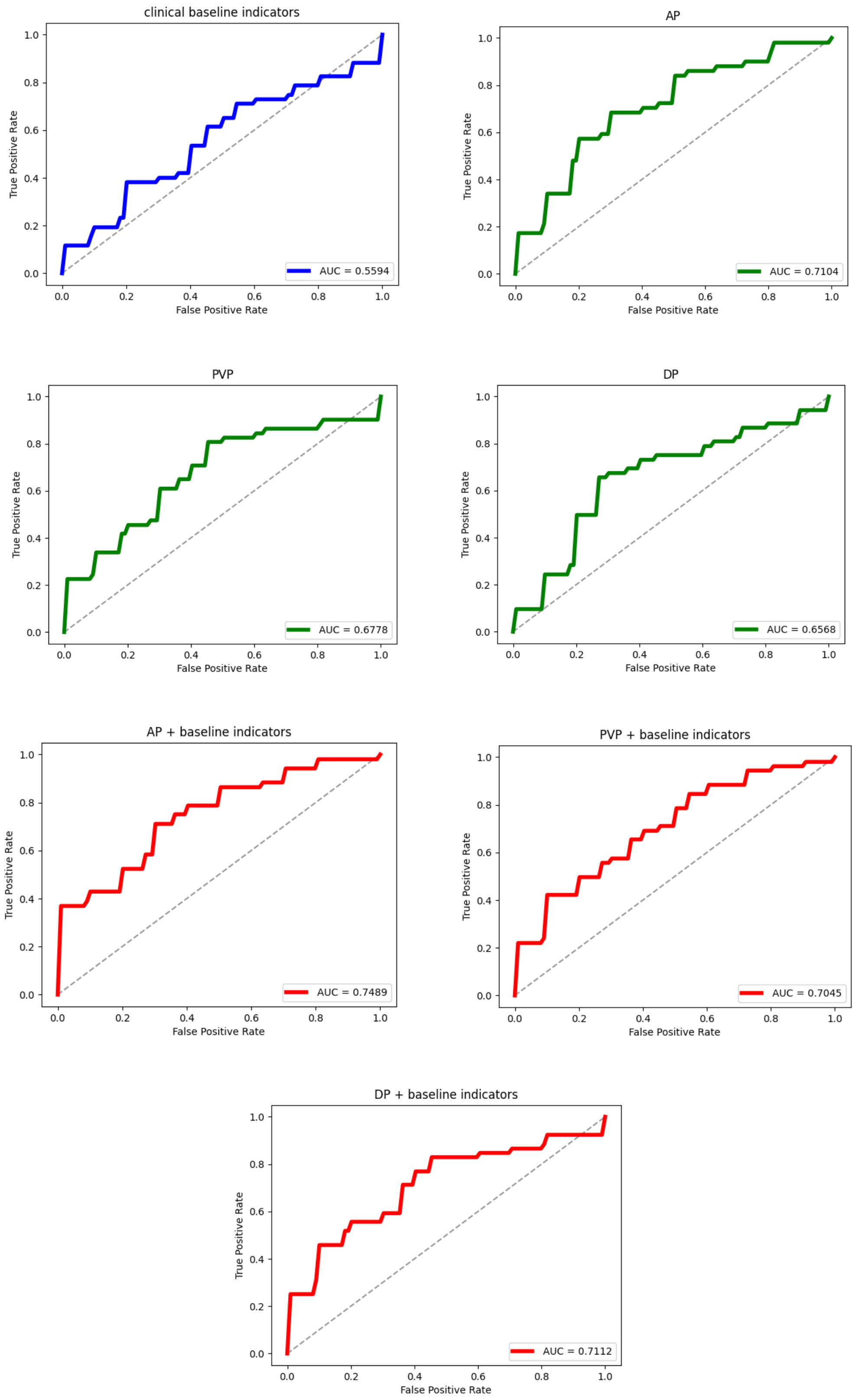

Fig 2: Results of using 7 feature representations in the XGBoost model

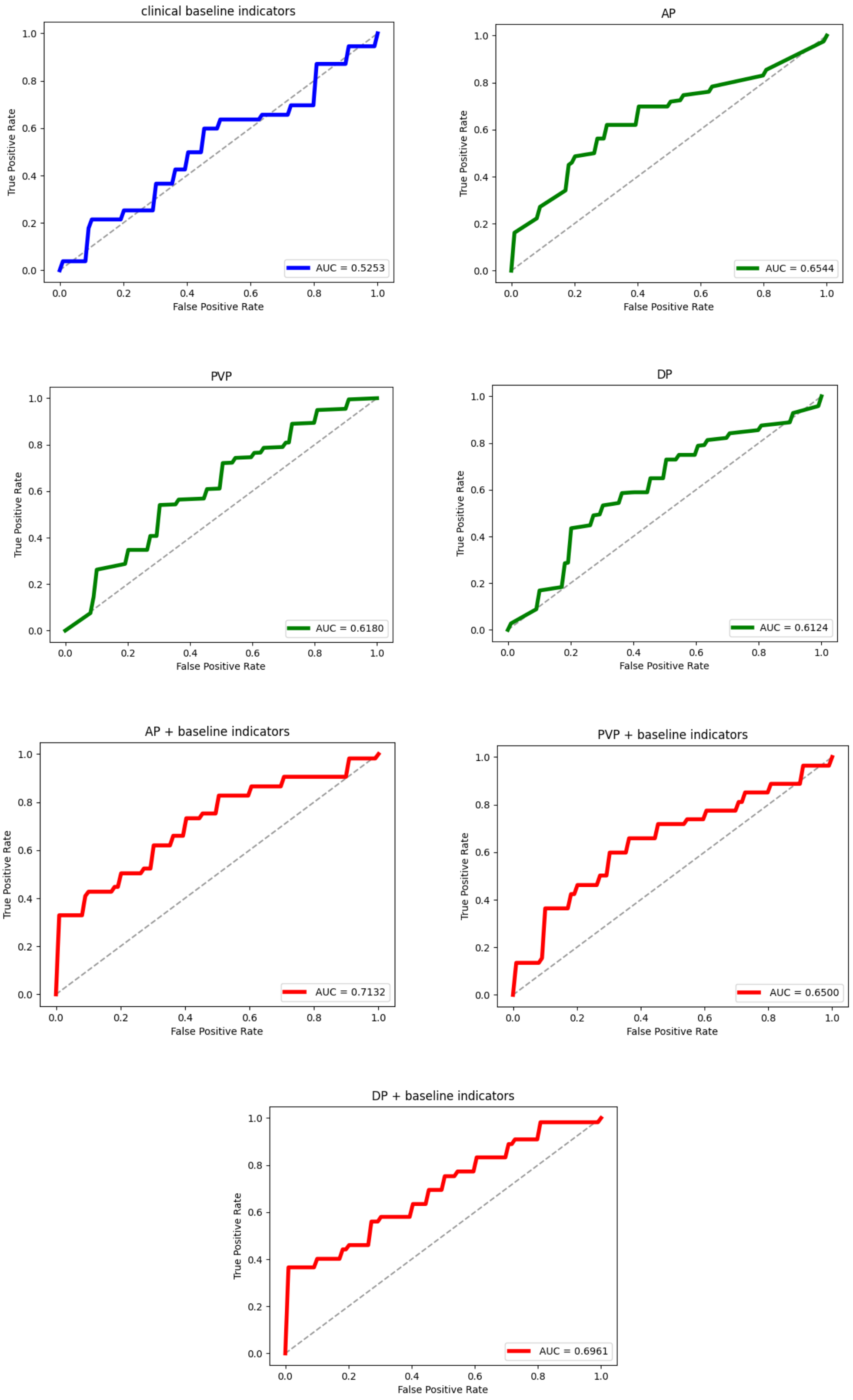

Fig 3: Results of using 7 feature representations in the GBDT model

Tab1: Other results of using 7 feature representations in the Catboost model

feature	Acc(%)	recall(%)	prec(%)	f1(%)
AP+clinical				
avg	71.28	57.73	80.25	68.27
95%CI	11.37	8.24	16.77	11.10
DP+clinical				
avg	67.29	65.64	71.50	66.67
95%CI	6.74	10.48	10.90	5.72
PVP+clinical				
avg	64.43	59.82	66.73	61.10
95%CI	5.61	17.35	8.46	10.49
AP				
avg	69.29	62.00	77.14	67.05
95%CI	9.97	10.53	14.62	8.14
DP				
avg	63.38	61.46	64.26	62.16
95%CI	6.63	10.96	7.97	8.33
PVP				
avg	63.38	59.46	65.20	62.12
95%CI	9.26	7.07	11.32	8.91
clinical				
avg	56.76	48.54	58.69	51.69
95%CI	4.31	14.46	4.94	7.58

Tab2: Other results of using 7 feature representations in the XGBoost model

feature	acc(%)	recall(%)	prec(%)	f1(%)
AP+clinical				
avg	73.95	69.09	77.02	72.34
95%CI	9.59	12.86	10.57	10.96
DP+clinical				
avg	66.52	69.09	69.16	67.12
95%CI	10.69	15.06	15.32	10.94
PVP+clinical				
avg	67.29	61.82	71.62	65.16
95%CI	3.26	7.92	8.10	3.29
AP				
avg	66.19	59.27	68.61	62.65
95%CI	9.37	15.20	12.15	12.67
DP				
avg	63.52	61.64	64.63	61.44
95%CI	6.00	16.30	10.52	10.81
PVP				
avg	63.52	59.27	63.88	60.43
95%CI	6.55	15.55	7.13	11.55
clinical				
avg	54.86	59.64	56.23	57.18
95%CI	6.52	2.55	9.16	3.44

Tab3: Other results of using 7 feature representations in the GBDT model

feature	acc(%)	recall(%)	prec(%)	f1(%)
AP+clinical				
avg	64.29	62.00	67.85	63.32
95%CI	11.80	14.94	16.05	11.74
DP+clinical				
avg	58.52	57.64	57.80	57.12
95%CI	9.16	16.23	7.38	11.24
PVP+clinical				
avg	60.71	54.00	61.29	56.69
95%CI	6.80	14.46	7.17	10.92
AP				
avg	63.48	58.18	66.95	61.42
95%CI	8.93	11.13	10.76	8.28
DP				
avg	59.48	64.91	57.72	60.86
95%CI	9.57	14.76	8.81	11.35
PVP				
avg	56.71	47.64	59.36	50.92
95%CI	4.63	12.88	6.70	10.15
clinical				
avg	49.86	46.18	51.54	48.54
95%CI	15.50	12.60	17.63	14.76
